# Comparative Microbial Nitrogen Functional Gene Abundances in the Topsoil vs. Subsoil of Three Grassland Habitats in Northern China

**DOI:** 10.3389/fpls.2021.792002

**Published:** 2022-01-14

**Authors:** Yuqing Liu, Qiaodong Chi, Hui Cheng, Huanxin Ding, Teng Wen, Jun Zhao, Xiaojuan Feng, Jinbo Zhang, Zucong Cai, Guohua Liu

**Affiliations:** ^1^State Key Laboratory of Urban and Regional Ecology, Research Center for Eco-Environmental Sciences, Chinese Academy of Sciences (CAS), Beijing, China; ^2^College of Resources and Environment, University of Chinese Academy of Sciences (CAS), Beijing, China; ^3^School of Geography, Nanjing Normal University, Nanjing, China; ^4^State Key Laboratory of Vegetation and Environmental Change, Institute of Botany, Chinese Academy of Sciences (CAS), Beijing, China; ^5^Jiangsu Center for Collaborative Innovation in Geographical Information Resource Development and Application, Nanjing, China; ^6^Zhongke Clean Soil (Guangzhou) Technology Service Co., Ltd., Guangzhou, China; ^7^Suzhou Station of Farmland Quality Protection, Suzhou, China; ^8^Key Laboratory of Virtual Geographical Environment, Ministry of Education, Nanjing Normal University, Nanjing, China

**Keywords:** grassland ecosystem, nitrogen functional gene, soil depth, real-time PCR, abundance

## Abstract

The microbial groups of nitrogen fixers, ammonia oxidizers, and denitrifiers play vital roles in driving the nitrogen cycle in grassland ecosystems. However, the understanding of the abundance and distribution of these functional microorganisms as well as their driving factors were limited mainly to topsoil. In this study, the abundances of nitrogen functional genes (NFGs) involved in nitrogen fixation (*nifH*), ammonia oxidation (*amoA*), and denitrification (*nirK*, *nirS*, and *nosZ*) were investigated in both topsoil (0–10 cm, soil layer with concentrated root) and subsoil (30–40 cm, soil layer with spare root) of three grassland habitats in northern China. The abundance of NFGs decreased with soil depth except for the archaeal *amoA* gene and the distribution of *nifH*, archaeal *amoA*, *nirK*, and *nirS* gene was significantly impacted by grassland habitats. Moreover, the distribution of NFGs was more responsive to the vertical difference than horizontal spatial heterogeneity. Redundancy analysis revealed that the distribution pattern of overall NFGs was regulated by grassland habitats, and these regulations were more obvious in the subsoil than in the topsoil. Variance partitioning analysis further indicated that soil resource supply (e.g., organic matter) may control the vertical distribution of NFGs. Taken together, the findings in this study could fundamentally improve our understanding of the distribution of N cycling-associated microorganisms across a vertical scale, which would be useful for predicting the soil N availability and guiding the soil N management in grassland ecosystems.

## Introduction

Nitrogen (N) cycling is considered a vital biogeochemical cycle on Earth as it controls the availability of nitrogenous nutrients and biological productivity in natural ecosystems (Vitousek and Howarth, 1991; Nelson et al., 2016). The underlying microbe-mediated functions are associated with the availability of nitrogenous nutrient supply and, thus, influence plant-based primary production ecosystems (Wang H. et al., 2017; Song et al., 2020; Wang et al., 2021; Zhou et al., 2021). A better understanding of the distribution of N functional microbes remains constrained.

Soil N cycle serves as an important component of the global N cycle, and the transformation among various N forms is largely accomplished by the enzymes encoded by several N functional genes (NFGs) harbored in diverse soil microbes (Robertson and Groffman, 2007; Hu et al., 2015). For instance, the nitrogen fixation (*nifH*) gene encodes the nitrogenase and catalyzes the conversion of N_2_ to NH_4_^+^, which, thereby, provides a primary source of N to natural ecosystems (Levy-Booth et al., 2014). Nitrification is a two-step process linking the reduced and oxidized forms of N, in which ammonia oxidation (*amoA*) is thought to be a rate-limiting step and mediated by the ammonia-oxidizing archaea (AOA) and ammonia-oxidizing bacteria (AOB) with the monooxygenase encoded by the gene *amoA* (Prosser and Nicol, 2008; Levy-Booth et al., 2014). Increasing evidence suggested that the NFGs abundances were correlated with the rate of the N cycling processes to some extent, and some of their ratios were considered as a useful indicator for the potential of N turnover capacity (Prosser and Nicol, 2012; Carey et al., 2016). Based on path analysis, AOA/AOB and nirK/S and nosZ gene abundances were the most important variables to predict nitrification and denitrification potential rates across terrestrial ecosystems (Xiong et al., 2012). Regional-scale experiments offered direct evidence of the relationship between N cycling process rate and N functional gene abundance (Chen et al., 2018). Moreover, the relative abundance of NFGs, such as AOA/AOB and (AOA + AOB)/(*nirK* + *nirS*) quantified as N turnover potential capacity, represented dominance in ammonia oxidization and NO_3_-N leaching, respectively (Tang et al., 2016, 2018). Therefore, better understanding the abundance and distribution of NFGs will offer an insight to know the soil N availability for a given natural ecosystem and thus to guide for predicting plant primary productivity.

Over the past few decades, spatial patterns and drivers of soil microbial communities have attracted extensive interest at various spatial scales (Fierer et al., 2010; Serna-Chavez et al., 2013; Fierer, 2017). These studies demonstrated that microbial taxa inhabiting varied surface soil habitats exhibit biogeographic patterns across broad geographical space (Chu et al., 2010; Brockett et al., 2012). It is believed that environmental factors, such as altitude, longitude, and latitude, usually covary with the changes in the geographical distance (Fierer and Jackson, 2006; Griffiths et al., 2011; Jia et al., 2017). Such horizontal differences are often characterized by heterogeneity of soil type, climate, vegetation, etc., and, of course, pronouncedly shape the distribution of microorganisms (Zhang et al., 2006; Hu et al., 2014; Chen et al., 2018). Moreover, the geographical distribution of microbial populations was also driven by geographical isolation even with similar characteristics and common ancestry (O’Malley, 2008). A previous study found that there is habitat specificity for microbial diversity and is no considerable dispersal limitation in bacteria distribution at a large scale in China (Wang X. B. et al., 2017). Although these findings have drastically improved our understanding of the pattern of microbes, we are still not sure if there is a horizontal difference in N-cycling microbes over hundreds of kilometers. Such knowledge is needed to improve our understanding of the N transformation processes in the grassland ecosystem especially under the ground of global climate change.

It is well recognized that both the biotic and abiotic factors could impact the abundance and distribution of N-cycling microbial communities (Hayden et al., 2010; Stone et al., 2015; Chen et al., 2018). For instance, Wei et al. (2017) and Cheng et al. (2019) exhibited that plant traits such as plant type, species richness, and biomass were important predictors of abundance and activity of ammonia-oxidizing and denitrifying communities in the grassland ecosystem. In contrast, numerous studies indicated that abiotic factors such as precipitation, soil pH, and organic carbon were pivotal to the proliferation of N-cycling microorganisms (Chen et al., 2015; Barrett et al., 2016; Zhou et al., 2021). Moreover, abiotic factors, especially precipitation, could indirectly affect the soil N-cycling microbes by impacting the accumulation of grassland biomass (Prober et al., 2015; Wang X. B. et al., 2017). However, these reports are restricted to the N-cycling microbes residing in near-surface soil while there was still less knowledge about their counterpart microbes inhabiting in deeper soil depths. Especially, Wakelin et al. (2008) provided evidence where soil C is strongly linked to diazotrophic ecology, pointing out that the quality and quantity of soil C influence the N-fixing bacteria. In addition, Chen et al. (2018) exhibited that abiotic and plant factors greatly predict the abundance and activity of ammonia-oxidizing and denitrifying communities across the Qinghai-Tibetan plateau.

Soil depth is regarded as an important factor in determining the distribution of microbial communities because of the environmental gradients along with the soil vertical profiles (Bai et al., 2017). Particularly, the oxygen level, moisture, nutrient availability, etc., sharply decrease with soil depth, and the soil layer of >30 cm is commonly characterized as the oligotrophic environment (Whitman et al., 1998; Eilers et al., 2012). Especially, more than 80% of roots distribute in the top 30 cm of soil in the grassland ecosystem (Yang et al., 2010). In fact, root vertical distribution could impact the microbial populations between soil depths mainly by providing different quantity and type of organic carbons and nutrients within a soil profile via root exudates and/or root litter decomposition (Peng et al., 2017; Shi et al., 2018). Therefore, the microbial biomass and activity considerably declined with the increasing depths as a consequence, which might lead to a reduction in population size as well as changes in the composition and diversity of microbial communities (Babujia et al., 2010; Bai et al., 2015). For example, researchers observed that both AOA and AOB rapidly decreased between 0 and 30 cm depth and then tended to mildly reduce below 30 cm soil depth (Banning et al., 2015; Tang et al., 2018). It has been widely accepted that AOA is preferred to adaptation in poor nutrient conditions and low soil pH environments (Prosser and Nicol, 2012), making the AOA less responsive to soil depth (Wang et al., 2014). In addition, different taxa might respond dissimilarly to the changes in abiotic factors along with soil profile (Tang et al., 2018). Until present, we still lack a comprehensive understanding of the N-turnover functional gene in the grassland ecosystem, which impedes our ability to predict the key services provided by the grassland ecosystem.

Grasslands are the dominant landscapes in China, which cover 41.7% of the national territory and are mainly distributed in the northern temperate zone, ranging from temperate grasslands to alpine grasslands (Kang et al., 2007). To control the precipitation variable, sampling sites were designed along the 400 mm isohyet in the grasslands of northern China to evaluate the distribution difference and N-transformation potential in the grasslands of Northern China (Wang et al., 2018). In this study, we use quantitative polymerase chain reaction of samples taken at two soil depths, namely, topsoil at 0–10 cm depth and subsoil at 30–40 cm depth from the representative steppe, and compare the difference between soil depths over hundreds of kilometers. In this study, we use the ratio of *nifH*/*nosZ* and (AOA + AOB)/(*nirK* + *nirS*) as the proxy of potential microbial N storage and NO_3_^–^-N leaching. We hypothesized that the abundance of NFGs varied with geographical distance and soil depth. It is expected that vertical difference and horizontal geographical differences in environmental factors would shape the distinct N-cycling microbial community. We also hypothesized that the major driving factors shifted from topsoil to subsoil, and we expected that soil resources prevailed over soil edaphic parameters in driving the variation of N-cycling microbes residing in the subsoil.

## Materials and Methods

### Site Description and Soil Sample Collection

**FIGURE 1:**
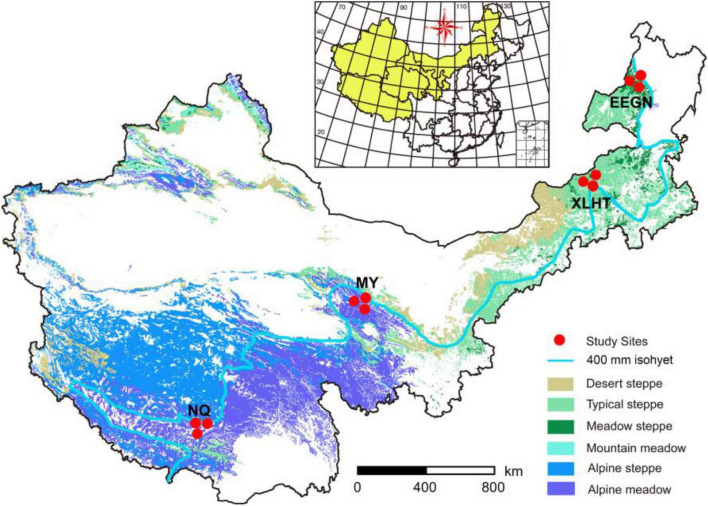
Locations of four selected grassland sites along 400 mm isohyet from the inner Mongolian Plateau and Qinghai-Tibet Plateau in China. The site abbreviations are defined in Table 1.

Four study sites with three representative grassland habitat types were located in the transition zone along 400 mm isohyet from the meadow steppe, typical steppe to the alpine meadow (Figure 1). Two sites were located on the Inner Mongolian Plateau, with meadow steppe dominated by *Leymus chinensis* in Eerguna [called EEGN at 520 m average sea level (a.s.l.)] and the other was typical steppe dominated by *Stipa grandis* in Xilinhot (called XLHT at 1250 m a.s.l.). The other two sites, namely Menyuan (MY, 3200 m a.s.l.) and Naqu (NQ, 4611 m a.s.l.), were located on the Qinghai-Tibet Plateau with the alpine meadow dominated by *Kobresia humilis* and *Potentilla nivea*. The aboveground biomass in the EEGN, XLHT, MY, and NQ sites was 112, 103, 350, and 116 g m^–2^, respectively; belowground biomass was 711, 2512, 3000, and 1509 g m^–2^, respectively (Table 1). According to the root distribution characteristics of plants, we collected soil samples from a depth of 0–10 cm (topsoil) and 30–40 cm (subsoil) at each study site in the plant growing season in September 2016. At each study site, five 1 m × 1 m subplots were established, one at each corner and one in the center of a 10 m × 10 m plot. The soil samples collected from the same layer at each plot were mixed into single composite samples, which were passed through 2 mm sieves and removed root materials. In total, 24 sieved soils (4 study sites × 3 replicates × 2 soil depths) were processed and stored as appropriate for physicochemical, biological, and molecular analyses.

**TABLE 1:** Characterization information of the natural grassland sites selected in this study.

Site^§^	Latitude	Longitude	Altitude (m)	MAP^a^ (mm)	MAT^b^ (°C)	Habitat type	Dominant plant species	Biomass (g m^–2^)*		Soil type
								Aboveground	Belowground	
EEGN	50°12′N	119°30′E	520	370	−2.14	Meadow steppe	*Leymus chinensis*	112	711	Chernozem
XLHT	43°38′N	116°42′E	1250	342	1.68	Typical steppe	*Stipa grandis*	103	2512	Calcicorthic aridisol
MY	37°37′N	101°19′E	3200	458	−0.78	Alpine meadow	*Kobresia humilis*, *Potentilla nivea*	350	3000	Alpine meadow soil
NQ	31°39′N	92°01′E	4611	405	−1.41	Alpine meadow	*Kobresia humilis*, *Potentilla nivea*	116	1509	Mollic-cryic cambisol

*^a^MAP, mean annual precipitation.*

*^b^MAT, mean annual temperature; data from https://www.worldclim.org/.*

*Estimated from its corresponding aboveground biomass according to Yang et al. (2010).

*^§^Sites: EEGN, Eerguna; XLHT, Xilinot; MY, Menyuan; NQ, Naqu.*

### Soil Physicochemical Analyses

Soil pH was measured using a glass electrode meter (S200 K, Mettler-Toledo International Inc., Shanghai, China) with a soil/water ratio of 1:2.5. Soil organic matter (OM) content was determined by H_2_SO_4_-K_2_Cr_2_O_7_ oxidation, while the total N (TN) content was analyzed by semimicro Kjeldahl digestion. Dissolved organic carbon (DOC) was measured in a 4:1 water/soil extract using an Analyzer Multi N/C (Analytic Jena, Jena, Germany). Soil NH_4_^+^-N and NO_3_^–^-N were extracted using 2 M KCl with a soil/solution ratio of 1:5 and measured using a continuous-flow analyzer (Skalar, Breda, Netherlands). The moisture content was determined gravimetrically by oven drying at 105°C to constant mass.

### DNA Extraction and Real-Time PCR Assay

Soil genomic DNA was extracted, quality-controlled, and quantified as described previously by Zhao et al. (2018). After extraction, the DNA samples were stored at −20°C for subsequent molecular analysis. Real-time PCR was performed on a CFX-96 thermocycler (Bio-Rad Laboratories Inc., CA, United States) to enumerate the abundances of microbial NFGs that involved in nitrogen fixation (*nifH*), ammonia oxidation (archaeal *amoA* and bacterial *amoA*), and denitrification (*nirK*, *nirS*, and *nosZ*) using the primer pairs listed in Table 2. The reaction mixture contained 10 μl of *Premix Ex* Taq™ (Takara Bio Inc., Kyoto, Japan), 2 μl of DNA extract (20 ng μl^–1^), 1 μl of each primer (10 μM), and 6 μl of sterile distilled water. The PCR procedure consisted of 2 min at 95°C, followed by 39 cycles of 10 s at 95°C, 20 s at 58°C, and 30 s at 72°C. Standard curves were plotted using 10-fold serial dilutions of the plasmid DNA inserted with the fragment of interest, following a previously established procedure (Zhao et al., 2016), and the amplification efficiencies ranged from 88.3 to 97.0% for different genes. The melting curve analysis was performed to ensure the amplification specificity, and the copy numbers were log_10_-transformed to normalize the values prior to statistical analysis.

**TABLE 2:** Summary of the genes investigated, the enzymes they encoded, and their functions in the nitrogen cycle.

Target gene	Primer set	Primer sequence (5′–3′)	Enzyme	Process	References
*nifH*	PolF	TGCGA**Y**CC**S**AA**R**GC**B**GACTC	Nitrogenase	Nitrogen fixation (N_2_-NH_4_^+^)	Poly et al., 2001
	PolR	AT**S**GCCATCAT**Y**TC**R**CCGGA			
Archaeal *amoA*	Arch-amoAF	**S**TAATGGTCTGGCTTAGACG	α subunit of ammonia monooxygenase	Ammonia oxidation (NH_4_^+^-NO_2_^–^)	Francis et al., 2005
	Arch-amoAR	GCGGCCATCCATCTGTATGT			
Bacterial *amoA*	AmoA1F*	GGGG**H**TT**Y**TACTGGTGGT	α subunit of ammonia monooxygenase	Ammonia oxidation (NH_4_^+^-NO_2_^–^)	Stephen et al., 1998
	AmoA2R	CCCCTC**K**G**S**AAAGCCTTCTTC			
*nirS*	Cd3aF	GT**S**AACGT**S**AAGGA**R**AC**S**GG	Nitrite reductase	Denitrification (NO_2_^–^-NO)	Throbäck et al., 2004
	R3cd	GA**S**TTCGG**R**TG**S**GTCTTGA			
*nirK*	nirK1F	GG**M**ATGGT**K**CC**S**TGGCA	Nitrite reductase	Denitrification (NO_2_^–^-NO)	Braker et al., 1998
	nirK5R	GCCTCGATCAG**R**TT**R**TGG			
*nosZ*	nosZFb	AACGCCTA**Y**AC**S**AC**S**CTGTTC	Nitrous oxide reductase	Denitrification (N_2_O-N_2_)	Rosch and Bothe, 2005
	nosZRb	TCCATGTGCAG**N**GC**R**TGGCAGAA			

*Boldface letters denote degenerate positions. M = A/C; R = A/G; S = G/C; Y = C/T; K = G/T; B = G/C/T; H = A/C/T; N = A/G/C/T.

### Statistical Analyses

The PASW Statistics 18 (SPSS Inc., Chicago, United States) and R (version 3.3.1) (R Core Team, 2012) were used to perform statistical analysis, and *P*-value of <0.05 was considered statistically significant. Data were tested for normality (Shapiro–Wilk normality test) and homogeneity of variance and were logarithm transformed when necessary to meet the criteria for a normal distribution. Multiple analysis of variance (MANOVA) was used to determine the effects of geographical location and soil depth on soil properties and NFGs abundance. For a given soil depth, the significant difference among sampling sites was tested using one-way ANOVA followed by LSD *post hoc* test. For a given site, two-tailed unpaired *t*-tests were used to compare each measurement between soil depths. The abundance of overall NFGs and soil properties was visualized with redundancy analysis (RDA). The Monte Carlo test (999 permutations) was further used to assess the effects of edaphic factors on the distribution of NFGs. Variance partitioning analysis was also performed to determine the relative contributions of soil edaphic parameters (soil pH and moisture) and soil sources (OM, DOC, TN, and NH_4_^+^-N content) on the variation in NFGs distribution in both soil depths.

## Results

### Soil Physicochemical Properties

Overall, physicochemical properties of the topsoil or subsoil differed significantly (*P* < 0.05) among the selected grassland sites except for the soil NO_3_^–^-N content in the subsoil. However, the different physicochemical properties varied among these four sampling sites at both topsoil and subsoil (Table 3). For instance, soil OM, TN, and NH_4_^+^-N contents were highest in the topsoil of the MY site, followed by the NQ, EEGN, and XLHT sites, while the highest NO_3_^–^-N content was observed in the EEGN site, followed by the NQ, XLHT, and MY sites. In the subsoil, soil OM and TN content were highest in the MY site, followed by the EEGN, XLHT, and NQ sites, whereas the highest NH_4_^+^-N and NO_3_^–^-N contents were observed in the EEGN site, with the lowest value being in the XLHT and MY sites, respectively (Table 3). Moreover, most tested soil physicochemical properties showed a decreasing trend from topsoil to the subsoil, whereas soil pH increased (*P* < 0.05) with the soil depth, except for the EEGN site (Table 3). The results of MANOVA showed that all soil edaphic properties were affected (*P* < 0.05) by geographical location, soil depth, and the geographical location × soil depth interaction term except for the soil DOC content (Table 4).

**TABLE 3:** Physicochemical characteristics of soils from different natural grassland sites and soil depths.

Physicochemical measurements	Sampling site^§^ and soil depth							
	Topsoil				Subsoil			
	EEGN	XLHT	MY	NQ	EEGN	XLHT	MY	NQ
pH	6.70 ± 0.07 b(a)	7.41 ± 0.18 a(b)	7.63 ± 0.15 a(b)	6.66 ± 0.09 b(b)	6.78 ± 0.17 b(a)	8.23 ± 0.02 a(a)	8.11 ± 0.05 a(a)	7.22 ± 0.02 b(a)
Moisture (%)	21.0 ± 0.7 b(a)	13.0 ± 0.5 d(a)	29.8 ± 2.8 a(a)	17.6 ± 0.5 c(a)	13.6 ± 2.6 b(b)	6.2 ± 1.0 c(b)	24.2 ± 0.4 a(a)	6.9 ± 0.3 c(b)
OM (g kg^–1^)	70.1 ± 3.7 c(a)	49.1 ± 10.5 d(a)	151.7 ± 15.2 a(a)	114.9 ± 3.4 b(a)	40.7 ± 0.8 b(b)	28.2 ± 2.8 c(b)	47.3 ± 5.8 a(b)	13.1 ± 0.0 d(b)
DOC (mg kg^–1^)	477.3 ± 26.7 b(a)	602.9 ± 196.8 ab(a)	968.7 ± 291.5 a(a)	590.7 ± 167.2 b(a)	121.6 ± 8.5 d(b)	344.6 ± 38.4 b(a)	455.8 ± 31.9 a(b)	193.5 ± 14.7 c(b)
Total N (g kg^–1^)	3.00 ± 0.11 c(a)	2.69 ± 0.47 c(a)	7.07 ± 0.86 a(a)	4.81 ± 0.37 b(a)	1.62 ± 0.12 b(b)	1.52 ± 0.11 b(b)	2.65 ± 0.34 a(b)	0.77 ± 0.00 c(b)
C/N	13.6 ± 0.4 a(a)	10.5 ± 0.4 b(a)	12.5 ± 1.6 a(a)	13.9 ± 0.9 a(a)	14.6 ± 0.7 a(a)	10.8 ± 0.3 b(a)	10.3 ± 0.1 bc(a)	9.9 ± 0.0 c(b)
NH_4_^+^-N (mg kg^–1^)	2.05 ± 0.14 c(a)	1.84 ± 0.26 c(a)	3.17 ± 0.12 a(a)	2.61 ± 0.42 b(a)	1.98 ± 0.06 a(a)	1.67 ± 0.14 b(a)	1.87 ± 0.12 ab(b)	1.77 ± 0.22 ab(b)
NO_3_^–^-N (mg kg^–1^)	45.0 ± 3.2 a(a)	20.2 ± 6.1 c(a)	5.6 ± 0.4 d(a)	36.6 ± 2.3 b(a)	14.4 ± 9.2 a(b)	9.0 ± 1.9 a(b)	5.5 ± 0.1 a(a)	6.6 ± 2.5 a(b)

*OM, organic matter; DOC, dissolved organic carbon. Values are means ± SD (n = 3). Different letters outside brackets represent significant differences within the same soil layer of the four sites at P < 0.05 according to LSD post hoc test, and different letters within brackets represent significant differences between two layers in the same site at P < 0.05 according to Student’s t-test. ^§^ The site abbreviations are defined in Table 1.*

**TABLE 4:** Overall effects of the sampling site and soil depth on the soil physicochemical characteristics analyzed by two-way ANOVA.

	Sites (*df* = 3)		Soil depth (*df* = 1)		Sites × depth (*df* = 3)	
	MS	*F*	MS	*F*	MS	*F*
pH (H_2_O)	2.1	165.9***	1.4	113.2***	0.1	11.7***
Moisture	0.03	168.0***	0.04	169.3***	0.001	3.5*
OM	39.5	77.5***	246.3	483.3***	30.6	60.0***
DOC	187,677.5	9.7***	871,110.8	44.9***	16,673.0	0.9
Total N	9.6	61.6***	45.3	291.9***	4.4	28.3***
C/N	12.9	28.6***	9.0	19.9***	8.0	17.8***
NH_4_^+^-N	0.6	13.8***	2.1	46.5***	0.5	11.2***
NO_3_^–^-N	634.2	34.3***	1946.9	105.3***	335.0	18.1***

df, degrees of freedom; MS, mean square; F, variance ratio. *Means P < 0.05; ***means P < 0.001.

### Abundance of Nitrogen Fixers and Ammonia Oxidizers

**FIGURE 2:**
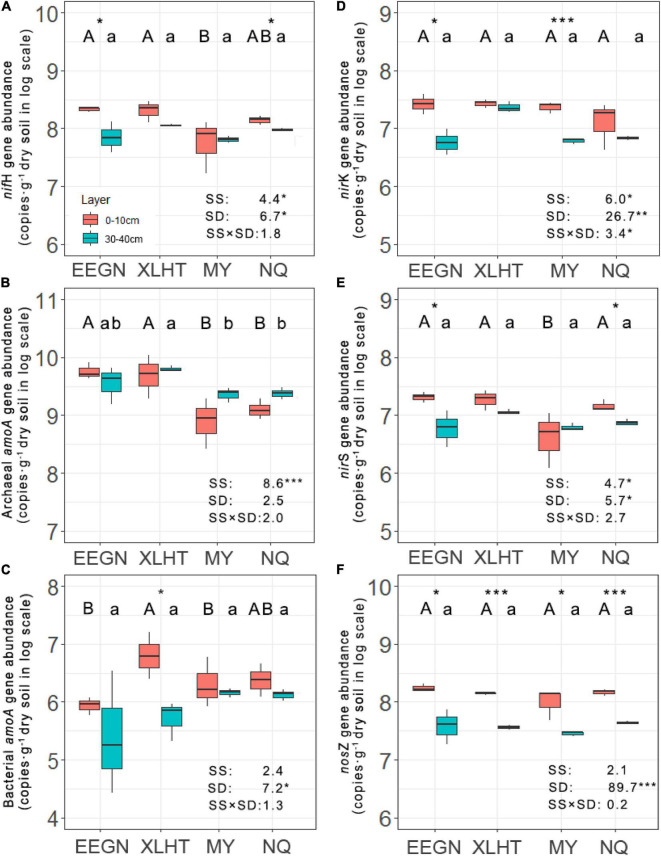
Abundances of *nifH* **(A)**, archaeal *amoA* **(B)**, bacterial *amoA* **(C)**, *nirK* **(D)**, *nirS* **(E)**, and *nosZ* **(F)** genes at soil depths of 0–10 and 30–40 cm at four grassland sites. Different uppercase and lowercase letters above the boxes indicate significant differences at *P* < 0.05 between sites according to LSD *post hoc* test in the topsoil and subsoil, respectively. An asterisk above the boxes indicates a significantly different level at *P* < 0.05 according to the student *t*-test for a given site. An asterisk next to the number indicates a significantly different level at *P* < 0.05 according to multiple analyses of variance. SS, sampling site; SD, soil depth; SS × SD, sampling site × soil depth. *P < 0.05; **P < 0.01; ***P < 0.001. The site abbreviations are defined in Table 1.

The abundance of nitrogen fixers decreased with the increase in soil depth at all the grassland sites except for the MY site, ranging from 1.29 × 10^7^ to 3.01 × 10^8^ copies⋅g^–1^ dry soil, and significant differences between topsoil and subsoil were observed in the EEGN and NQ sites (Figure 2A). Moreover, the population of nitrogen fixers differed significantly among the sampling sites in the topsoil, being higher at the EEGN and XLHT sites as compared with the MY site, while no significant difference was observed in the subsoil. The distribution of nitrogen fixers was affected (*P* < 0.05) by the sampling site and soil depth (Figure 2A).

Unlike the results of nitrogen fixers, the population size of AOA increased with the increasing soil depth in all sampling sites except for the EEGN site, ranging from 6.16 × 10^8^ to 6.27 × 10^9^ copies⋅g^–1^ dry soil, although there was no significant difference between soil depth for a given site (Figure 2B). Furthermore, significant differences were observed among sampling sites in both topsoil and subsoil. For instance, the abundance of AOA was higher in the EEGN and XLHT sites than that in the MY and NQ sites in the topsoil. In contrast, the abundance of AOB showed a declining trend from topsoil to subsoil at all the grassland sites, ranging from 2.52 × 10^5^ to 6.29 × 10^6^ copies⋅g^–1^ dry soil (Figure 2C). A significant difference between soil depth was observed only in the XLHT site. The abundance of AOB changed considerably (*P* < 0.05) among the sampling sites in the topsoil, whereas there was no significant difference in the subsoil. In addition, MANOVA results revealed that the distribution of AOA was affected by the sampling site while the distribution of AOB was impacted by soil depth (Figures 2B,C).

### Abundance of Nitrite Reductase (*nirS* and *nirK*) and Nitrous Oxide Reductase (*nosZ*) Genes

The abundance of *nirK*- and *nirS*-type nitrite reductase gene shifted from 3.49 × 10^6^ to 3.85 × 10^7^ and from 1.01 × 10^6^ to 2.45 × 10^7^ copies⋅g^–1^ dry soil, respectively, in all soil samples. They consistently decreased from topsoil to subsoil in all the sampling sites except for *nirS*-type nitrite reductase gene at the MY site. These declines were significant (*P* < 0.05) in the EEGN and MY sites for *nirK*-type nitrite reductase gene and the EEGN and NQ sites for *nirS*-type nitrite reductase gene, respectively (Figures 2D,E). Significant differences in abundance were observed in the subsoil of *nirK*-type nitrite reductase gene and the topsoil of *nirS*-type nitrite reductase gene among the sampling sites. Both sampling sites and soil depth significantly affected the distributions of *nirK*- and *nirS*-type nitrite reductase gene (Figures 2D,E). The abundance of nitrous oxide reductase (*nosZ*) gene ranged from 1.81 × 10^7^ to 2.03 × 10^8^ copies g^–1^ dry soil in all soil samples and accounted for the largest proportion (43.9–88.4%) of denitrifies. It declined considerably (*P* < 0.05) with the increasing soil depth in all the grassland sites, whereas there were no significant differences among the sites for both soil layers (Figure 2F). Thus, only soil depth was the significant (*P* < 0.001) factor influencing the distribution of nitrous oxide reductase (*nosZ*) gene.

### Potential Nitrogen-Cycling Capacity

**FIGURE 3:**
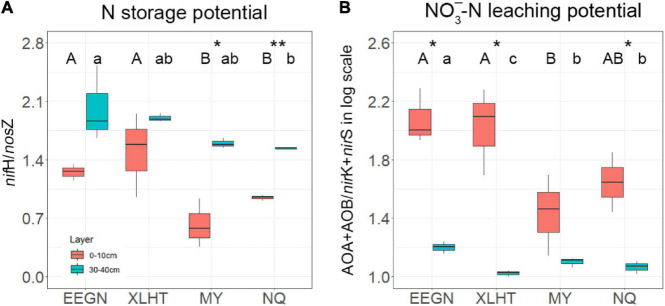
Microbial N storage potential **(A)** and nitrate leaching potential **(B)** at soil depths of 0–10 and 30–40 cm at four grassland sites. Values (means ± SD, *n* = 3) within the same soil depth followed by different letters are significantly different at *P* < 0.05 according to LSD *post hoc* test. An asterisk above the bars indicates a significantly different level at *P* < 0.05 according to the student *t*-test for a given sampling site. *P < 0.05; **P < 0.01. The site abbreviations are defined in Table 1.

The topsoil N-storage potential at the EEGN and XLHT sites was significantly (*P* < 0.05) higher than that at the MY and NQ sites. The N-storage potential increased with the increase in soil depth at all the grassland sites, and the significant differences between topsoil and subsoil were observed at the MY and NQ sites (Figure 3A). The NO_3_^–^-N leaching potential in the EEGN and XLHT topsoil was almost two times higher than that at the MY and NQ sites, and the NO_3_^–^-N leaching potential significantly (*P* < 0.05) decreased with soil depth at all the sites except for MY (Figure 3B).

### Relationships Between Overall Nitrogen Functional Genes and Soil Edaphic Factors

**FIGURE 4:**
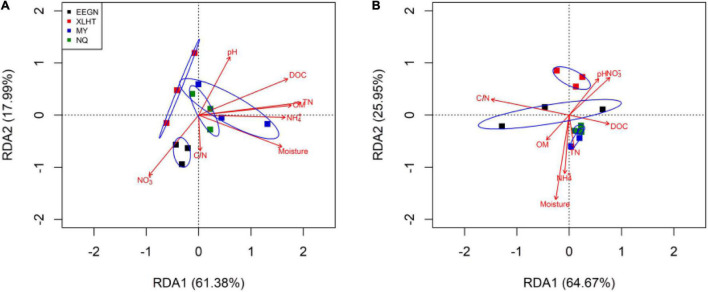
Redundancy analysis of overall nitrogen functional genes and soil characteristics for individual samples at a soil depth of 0–10 cm **(A)** and 30–40 cm **(B)**, respectively. The site abbreviations are defined in Table 1.

**FIGURE 5:**
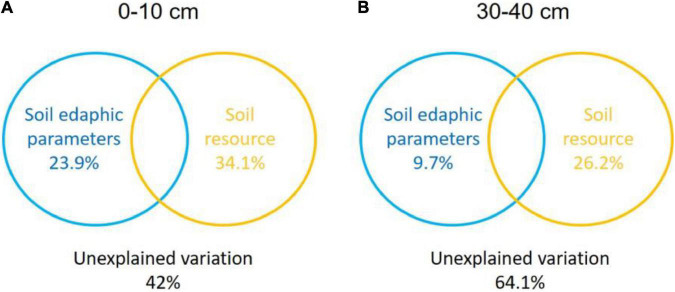
Variance partitioning analysis (VPA) of the effects of soil physical parameter and soil resource supply on the variation in the NFGs distribution at a soil depth of 0–10 cm **(A)** and 30–40 cm **(B)**. Circles on the edges of the triangle show the percentage of variation explained by each variable.

The Monte Carlo permutation test showed a significant (*F* = 2.71, *P* = 0.004) and positive correlation between the ordination of overall NFGs and soil edaphic properties. The RDA biplot revealed that the ordination of the overall NFGs was separated from the soil layers in the first axis, and the pattern of ordination in the topsoil was considerably distinct from that in the subsoil (Figure 4 and Supplementary Figure 1). In topsoil, the ordinations of overall NFGs at the MY and NQ sites were similar, with clear separation from that at the EEGN sites and a small overlap in the XLHT site (Figure 4A). The differences in the ordination of overall NFGs were enlarged in the subsoil. Especially, the ordinations of NFGs at the MY and NQ sites were grouped together, and they were distinctly separated from that at the EEGN and XLHT sites, with the EEGN and XLHT sites being separated from each other (Figure 4B). The effect of individual soil factors on the ordination of NFGs is shown by the direction and the length of the vectors. Soil moisture, OM, DOC, TN, and NH_4_^+^-N contents were closely (*P* < 0.05) correlated with the ordination of the overall NFGs in topsoil, whereas no significant correlations were observed between the NFGs ordination and individual soil property in the subsoil (Figure 4 and Supplementary Figure 1). Variance partitioning analysis showed the relative contributions of soil physical parameters and soil resource supply on NFGs distribution (Figure 4). The soil edaphic parameters and soil resource variables explained 23.9 and 34.1% of the observed variation in NFGs distribution in the topsoil, while these variables explained 9.7 and 26.2% of the observed variation, respectively (Figure 5).

## Discussion

### Changes in the Distribution of Nitrogen-Cycling Microbes Across Three Grassland Habitats

We assumed that the effect of the geographical distance on N-cycling microorganisms was embodied in the effects of temperature and/or precipitation (Griffiths et al., 2011; Brockett et al., 2012; Yang et al., 2014). As a result, our experiment was designed along with 400 mm isohyet and thus eliminated differences in the environmental factors relating to geographical location (Jia et al., 2017). In this study, geographical location considerably impacted the distribution of N-fixer, AOA, *nirK*-type, and *nirS*-type denitrifiers, suggesting that a geographical distance over hundreds of kilometers might influence the distribution pattern of N-cycling microorganisms (De Vries et al., 2012; Chu et al., 2016). This result might be attributed to heterogeneous climate, vegetation, and soil type caused by geographical distances between every pair of sites ranging from 762 to 3069 km (Supplementary Table 1). As per the principle of microbial biogeography, everything is everywhere, but the environment selects (de Wit and Bouvier, 2006), distribution of free-living microbial taxa exhibited dissimilarity with increasing geographical distance (Chu et al., 2016; Azziz et al., 2017; Escalas et al., 2021). The AOA gene abundance was significantly influenced by the geomorphic zones at the landscape scale (Hayden et al., 2010; Song et al., 2020; Zhou et al., 2021), which was consistent with this study. Previous studies also found that soil type exerted an effect on the abundance of the *nirK*-type and *nirS*-type denitrifies from two geographical zones (Azziz et al., 2017). Notably, the abundance of AOB and *nosZ* genes was less responsive to geographical location (Figure 2), suggesting that dispersal limitation might slightly influence the distribution of AOB and *nosZ*-type denitrifier in the study area (Chu et al., 2016; Wang X. B. et al., 2017). The ratio of *nifH*/*nosZ* and (AOA + AOB)/(*nirK* + *nirS*) in the EEGN and XLHT topsoil is significantly higher than that in the MY and NQ topsoil, suggesting that the temperate meadow might have a higher potential N fixation and NO_3_^–^-N leaching than alpine meadow (Tang et al., 2018). This result might be explained by the fact that microbes related energy-consume N process habitat preference and, thus, microbes in an alpine meadow with lower temperature may limit N fixation and NO_3_^–^-N leaching capacity than that in temperature meadow (Chen et al., 2018; Frindte et al., 2019).

### Shifts in the Abundance of Nitrogen Functional Genes Between Soil Depths

In general, subsoil differed in a range of edaphic factors from topsoils (Fierer et al., 2003; Rumpel and Kögel-Knabner, 2011; Schnecker et al., 2015), such as soil pH, moisture, oxygen level, and nutrient availability and consequently led to a shift in the abundance of soil microorganisms (Eilers et al., 2012; Wang et al., 2014; Ying et al., 2017). Alternatively, the distribution of AOA was not affected by the soil depth, thus indicating that AOA preferred to adaptation in lower soil depth with poor nutrient condition (Prosser and Nicol, 2012; Sun et al., 2019; Wang et al., 2019). It is also likely that the root system of vegetation cannot reach a certain depth of soil, supporting a depth-related root disturbance on the soil microbial community (Haichar et al., 2008; Shi et al., 2018). The relatively low N-cycling gene in the alpine meadow might be attributed to the fact that alpine grasslands have a relatively much shallower root distribution than temperate grassland (Yang et al., 2010). These observations together indicate that the N processes and soil functionality decline along with soil profiles, which is consistent with the results of previous studies (Bai et al., 2017; Jones et al., 2018; Tang et al., 2018), demonstrating that the soil functionality was greatly affected by soil depths (Yao et al., 2018; Xu et al., 2020). Researchers have found that *nirK* and *nirS*-type denitrifiers preferred to reside in surface soil with high organic C (Bru et al., 2011; Petersen et al., 2012; Prosser and Nicol, 2012). Soil C source and C/N ratio decreased from topsoil with dense root to subsoil with spare root, resulting in the reduction of *nirK* and *nirS*-type denitrifiers, and thus decrease in NO_3_^–^-N leaching potential between soil depths (Fierer et al., 2003; Tang et al., 2018). However, an increase in N-storage potential with increasing soil depths was observed in the MY and NQ sites, which might be attributed to permafrost-affected deeper alpine meadow soil (Chen et al., 2018).

### Soil Resource Supply Drives the Distribution of Nitrogen Functional Genes

As shown by RDA, the abundances in NFGs at the NQ and MY sites were grouped together, and they were distinctly separated from the EEGN and MY sites, respectively, in the subsoil, suggesting their distinct N-cycling microbial pattern in the subsoil from kilometers apart. These results are supported by previous findings that the abundance and distribution of dominant taxonomic groups varied across habitats in the Chinese northern dryland (Wang X. B. et al., 2017). These findings together indicate that the distribution of N-cycling microbes is structured by habitat-associated vegetation type in the subsoil. In this study, the overall distribution of NFGs depicted a habitat-specific pattern, reflecting their ecological characteristics such as physiological capabilities or habitat preferences (Fierer et al., 2007; Pointing et al., 2009). We found that variation in the distribution of NFGs in the subsoil was primarily related to soil resource supply, but not to the soil edaphic parameters. The clear and broad consensus is that soil microbes are limited by soil resources including C and N availability (Fierer et al., 2003; Hu et al., 2014; Chen et al., 2016). Recently, Chen et al. (2018) examined the geographical pattern of the functional N gene using intensive soil sampling across the Tibetan alpine grassland and exhibited that soil resource was the main predictor of N-cycling microbial community, while plant traits affected them *via* altering the SOC availability (Li et al., 2017). Likewise, we observed that soil resource was an important predictor in the topsoil. Moreover, the soil resource explained more variation in N-cycling microbial community in deeper soil depth, indicating that the distribution of N-cycling microbes residing in the deeper soil depths was associated with soil resource supply (Yang et al., 2013; Liu et al., 2020). This result was supported by the finding that the vertical distribution of N-cycling microorganisms was largely attributed to the decline in soil C availability. Barrett et al. (2016) reported that the C amendment resulted in the increase of denitrifies abundance and this impact changed with soil depth due to the decline in C availability. In addition, the interaction of plant and edaphic factors in temperate grasslands affects the microbial community distribution and the effect depends on the soil depth (Yao et al., 2018). Hou et al. (2018) also presented direct evidence that root exudates can act as inducible C sources for heterotrophic denitrifying bacteria. Thus, the plant–soil interaction (i.e., rhizosphere effect and litter decomposition) involved in the distribution of NFGs should be further studied to reveal how the plant traits regulate the abundance of NFGs.

## Conclusion

In general, the abundance of NFGs gradually decreased with soil depth in the three grassland habitats. We also found that the distribution of free N-cycling microbial taxa exhibited dissimilarity at the landscape scale. In addition, NFGs distribution was more responsive to the vertical difference than horizontal spatial heterogeneity except for the archaeal *amoA* gene. The variation in N-transformation potential among the three grassland habitats may be connected with the energy-consuming N-cycling microorganism habitat preference. The distribution of overall NFGs exerted a habitat-specific pattern and the driving factors of NFGs shifted from the topsoil to the subsoil. Moreover, soil resource supply controlled the vertical distribution of NFGs rather than soil edaphic parameters.

### Uncertainties and Perspectives

The first uncertainty was the sampling rule along 400 mm isohyet, which neglects various environmental factors across the large geographical distance. It is also difficult to completely disentangle the vertical N-cycling ecological processes by only determining the NFGs abundance in limited sampling sites. Intensive sampling would be helpful to improve the understanding of active functional groups within the surface and subsoils and determine how these functional groups change with the soil profile. Second, we assume the copy number of a gene provides information about the potential N store and N release capacity of soil microorganisms in grassland, which may overestimate the nitrogen turnover potential. Besides, our research takes no account of other N-cycling processes, such as the mineralization of fungi. We do not provide a complete insight into measuring the capacity of microbial N turnover. Molecular analyses can be integrated with gene expression and rhizosphere isotopic tracing techniques to measure actual N storage and turnover potentials in future research.

## Data Availability Statement

The raw data supporting the conclusions of this article will be made available by the authors, without undue reservation.

## Author Contributions

JBZ, XF, JZ, and TW devised the study. YL carried out the experiments, analyzed the data, and wrote the first draft of the manuscript. QC, HC, and HD assisted with the experiments. All authors contributed to the preparation of the manuscript.

## Conflict of Interest

JZ, JBZ, and ZC were employed by the company Zhongke Clean Soil (Guangzhou) Technology Service Co., Ltd. The remaining authors declare that the research was conducted in the absence of any commercial or financial relationships that could be construed as a potential conflict of interest.

## Publisher’s Note

All claims expressed in this article are solely those of the authors and do not necessarily represent those of their affiliated organizations, or those of the publisher, the editors and the reviewers. Any product that may be evaluated in this article, or claim that may be made by its manufacturer, is not guaranteed or endorsed by the publisher.
