# Supplementary material for: Comparative Microbial Nitrogen Functional Gene Abundances in the Topsoil vs. Subsoil of Three Grassland Habitats in Northern China
Source: Front Plant Sci. 2022 Jan 14;12:792002. doi: 10.3389/fpls.2021.792002 (PMC8798409; doi:10.3389/fpls.2021.792002)

Figure. S1 Redundancy analysis of overall nitrogen functional genes and soil characteristics. The site abbreviations are defined in Table 1.

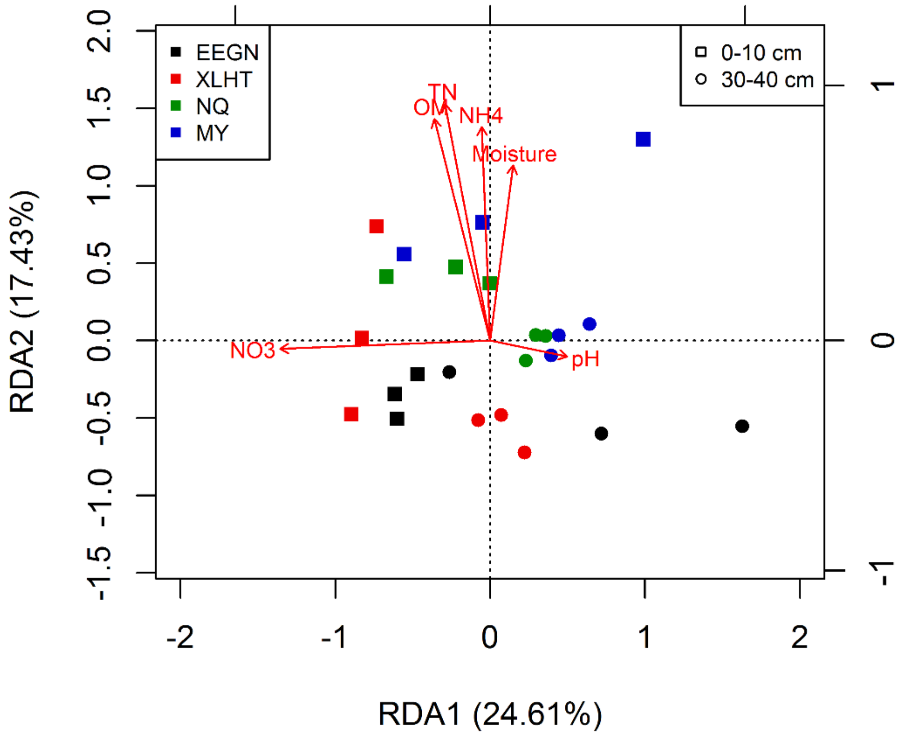

Supplement: Supplementary file 1 [file Image_1.pdf]
