# Supplementary material for: Comparative Microbial Nitrogen Functional Gene Abundances in the Topsoil vs. Subsoil of Three Grassland Habitats in Northern China
Source: Front Plant Sci. 2022 Jan 14;12:792002. doi: 10.3389/fpls.2021.792002 (PMC8798409; doi:10.3389/fpls.2021.792002)
Supplement: Supplementary file 2 [file Table_1.pdf]

Table S1. Pairwise geographical distance (km) between every pair of sites. The site abbreviations are defined in Table 1.

	EEGN	XLHT	NQ	MY
EEGN				
XLHT	762			
NQ	3069	2538		
MY	2011	1458	1081	

The geographical distance was calculated based on the latitude and longitude of each site.
